# Supplementary material for: Communicative modulations of early action components support the prediction of distal goals
Source: PLoS One. 2024 Jun 27;19(6):e0306072. doi: 10.1371/journal.pone.0306072 (PMC11210802; doi:10.1371/journal.pone.0306072)
Supplement: Attachment — Submitted filename: Response_to_reviewers.docx [file pone.0306072.s002.docx]

**Reviewer #1**: Overall, the authors did a good job of addressing the comments that I had in the first round. The manuscript reads much better now. I have a couple of points that came up during this reading that I think would make the manuscript much stronger in how the results are interpreted.

Lines 622-625: the authors interpret the non-significant second-movement type effect as demonstrating that participants do not need the velocity information of the second movement, and thus that “participants can rely on direct movement-to-goal simulation”. However, a non-significant p-value should not be taken as evidence for a lack of effect. Given that this outcome is important for the overall interpretation of the experimental effects, the authors should conduct an additional analysis to determine if there is indeed evidence for a lack of effect, for example using a Bayesian approach.

**We clarify this in the Discussion section of Experiment 1 (lines 677-686), where we now make more explicit our interpretation of our findings. More specifically, we conclude that the findings of Experiment 2, together with those of Experiment 1, are consistent with the possibility that *both* types of simulation (movement-to-movement and movement-to-goal) can play a role in how participants link early communicative modulations to distal goals.**

Experiment 3. On re-reading the discussion of these results, I wonder about the conclusion that the authors draw: “while the first movement still contained useful velocity information that could be used to simulate the distal goal, its change in direction led to a weakened relationship with the distal goal”. The authors acknowledge that the change in direction weakened the link between the first movement and the goal, but I wonder if it did so to the point that the connection becomes arbitrary. In a Gestalt framing of these experiments, the two movements are seen as one sequence or action chain due to their similarity in direction. But with this similarity in direction being removed, these two movements would not likely show the type of Gestalt coherence that would link them as being part of one goal-directed sequence. The seemingly arbitrary mapping between goal and first movement could be learned through perceptual experience, but not if there is no feedback. I don’t suggest that the authors add any additional experiments or anything to that extent, but I think that this (or a similar) interpretation should also be presented as a possible alternative.

**The purpose of our manipulation of direction in Exp 3 was indeed to weaken the relationship of the first movement with the second movement. However, our data suggest that this did not make the connection arbitrary: The majority of participants still chose a motor iconic mapping in the Sliding-Sliding condition (see Figure 10A)**

**Regarding the suggestion that a pure learning account could also explain our results: It is important to remember that, during trials, participants never saw the box actually being delivered to the near or far target. Learning by association, we think, could only be between first and second movement, but not the distal goal. As such, we would like to point towards our existing discussion of alternatives to a simulation account (lines 235-237), where we mention statistical learning as a theoretical possibility. We also now include Gestalt-like grouping principles (e.g., similarity) as a theoretical possibility.**

Minor:
Lines 803-807: this is quite a long sentence with a lot of information. I think it would be helpful to break this up a bit.

**We did this now (see lines 822-826).**

**Reviewer #2:** I thank Dockendorff et al. for thoroughly addressing the points I raised in my comments.

I still find a minor incongruence in Experiment 2. In lines 577-578, the authors reported that “However, there was no significant main effect of the first movement type.” Yet, in lines 646-648, they wrote, “This was indicated by the higher consistency and higher number of motor-iconic mappings in the Sliding-Jumping condition compared to the Jumping-Jumping condition.” Could the authors clarify this? Perhaps the authors should limit their conclusions to the mapping scores.

**We thank R2 for pointing out this incongruence. Indeed, there was no significant main effect of first movement type. We corrected this in the Discussion of Experiment 2 (lines 653-655), where we now limit our interpretation to motor-iconic mappings. We also point out that in the Sliding-Jumping condition consistency increased as a function of degrees of exaggeration.**

I appreciated that the authors included significance bars in the plots; however, this is limited to within-subject factors. I believe it would be relevant to include bars to highlight significant results on the between-subject factors as well, to communicate the findings better.

**We now added the significance bars across conditions whenever the differences between distributions are significant.**
